# An extracorporeal carbon dioxide removal (ECCO_2_R) device operating at hemodialysis blood flow rates

**DOI:** 10.1186/s40635-017-0154-1

**Published:** 2017-09-06

**Authors:** R. Garrett Jeffries, Laura Lund, Brian Frankowski, William J. Federspiel

**Affiliations:** 1 Department of Bioengineering, University of Pittsburgh, Pittsburgh, PA USA; 2 McGowan Institute for Regenerative Medicine, University of Pittsburgh, 3025 E Carson St, Suite 226, Pittsburgh, PA 15203 USA; 3 ALung Technologies, Inc., 2500 Jane Street, Suite 1, Pittsburgh, PA 15203 USA; 4 Department of Chemical Engineering, University of Pittsburgh, Pittsburgh, PA USA; 5 Department of Critical Care Medicine, University of Pittsburgh Medical Center, Pittsburgh, PA USA

**Keywords:** Extracorporeal carbon dioxide removal, CO_2_ removal, Artificial lung, Gas exchange, Chronic obstructive pulmonary disease, Acute respiratory distress syndrome

## Abstract

**Background:**

Extracorporeal carbon dioxide removal (ECCO_2_R) systems have gained clinical appeal as supplemental therapy in the treatment of acute and chronic respiratory injuries with low tidal volume or non-invasive ventilation. We have developed an ultra-low-flow ECCO_2_R device (ULFED) capable of operating at blood flows comparable to renal hemodialysis (250 mL/min). Comparable operating conditions allow use of minimally invasive dialysis cannulation strategies with potential for direct integration to existing dialysis circuitry.

**Methods:**

A carbon dioxide (CO_2_) removal device was fabricated with rotating impellers inside an annular hollow fiber membrane bundle to disrupt blood flow patterns and enhance gas exchange. In vitro gas exchange and hemolysis testing was conducted at hemodialysis blood flows (250 mL/min).

**Results:**

In vitro carbon dioxide removal rates up to 75 mL/min were achieved in blood at normocapnia (pCO_2_ = 45 mmHg). In vitro hemolysis (including cannula and blood pump) was comparable to a Medtronic Minimax oxygenator control loop using a time-of-therapy normalized index of hemolysis (0.19 ± 0.04 g/100 min versus 0.12 ± 0.01 g/100 min, *p* = 0.169).

**Conclusions:**

In vitro performance suggests a new ultra-low-flow extracorporeal CO_2_ removal device could be utilized for safe and effective CO_2_ removal at hemodialysis flow rates using simplified and minimally invasive connection strategies.

## Background

Mechanical ventilation has long been the standard of care for severe lung failure. A major and paradoxical complication of mechanical ventilation is direct trauma to already ailing lungs caused by over-distention and damage of alveolar tissue due to excessive positive pressures or volumes in the lung [1]. Extracorporeal membrane oxygenation, or ECMO, has more recently become recognized as a last resort option for severe lung failure when mechanical ventilation is failing or is not an alternative. Unlike mechanical ventilation, ECMO performs the function of blood oxygenation and carbon dioxide (CO_2_) removal independently of the lungs, allowing injured tissue to rest and heal [2]. ECMO is associated, however, with a higher risk of severe complications compared to mechanical ventilation because it requires full circulatory diversion of venous blood to achieve its intended function [3].

The primary complication risks of ECMO are associated with cannulation, exposure of blood to foreign materials, the concomitant requirement for systemic anticoagulation, and the stresses induced by mechanical pumping [4]. The degree of risk associated with these factors correlates with the extracorporeal blood flow rate necessary for treatment [4–6]. To provide full extracorporeal oxygenation of venous blood requires circuit flows up to the full cardiac output (4000–7000 mL/min) [5, 7]. In contrast, full metabolic CO_2_ removal can be achieved at much lower extracorporeal blood flows. CO_2_ is predominantly carried in the form of highly soluble bicarbonate ion that rapidly restores depleting CO_2_ as it is eliminated, and the CO_2_ dissociation curve is essentially linear and does not saturate like the oxyhemoglobin dissociation curve [8–10]. These differences also provide the opportunity to augment CO_2_ removal efficiency with gas exchanger design features aimed at reducing the thickness of the diffusive boundary layer at the gas exchange surface, where gas transport through blood is limited to diffusion [11].

The degree of risk associated with extracorporeal lung support is reduced when lower blood flows are needed to provide clinically meaningful benefit [5]. The ability to efficiently remove CO_2_ at lower blood flows has motivated use of extracorporeal CO_2_ removal, or ECCO_2_R, as an alternative or supplement to mechanical ventilation. The two primary clinical indications where this objective is feasible are acute exacerbations of chronic obstructive pulmonary disease (ae-COPD) and moderate to severe ARDS, where lung protective ventilation strategies are necessary but are unable to maintain safe levels of CO_2_ removal [12, 13]. ECCO_2_R was shown to reduce intubation rates in ae-COPD patients failing less-invasive ventilation and assisted in weaning from ventilation [14–19]. Hypercapnia was also managed in moderate ARDS patients using ECCO_2_R to facilitate more protective ventilation strategies by enabling reduction of tidal volumes to ≤ 4 mL/kg without complications [20, 21]. Associated risk remains the primary obstacle of ECCO_2_R adoption in these indications however. Currently approved ECCO_2_R systems can operate at blood flows around 500 mL/min, but still require cannula with size greater than 15 Fr [4]. The ability to provide the same levels of CO_2_ removal at even lower flows will enable the use of smaller catheters that are similar in size to commonly used dialysis catheters that are 9–14 Fr.

We are developing a next-generation ECCO_2_R device that operates at lower blood flows (250 mL/min) with minimal surface area and clinically significant CO_2_ removal rates. CO_2_ removal of 70–160 mL/min has been shown to benefit patients with hypercapnia, which translates to a target CO_2_ removal rate of ≥ 25–35% metabolic CO_2_ production (~ 200–250 mL/min) at normocapnia (pCO_2_ = 45 mmHg) [22, 23]. The device also should maintain an optimal degree of fluid washing around the hollow fiber membranes to eliminate regions of stagnation but without causing unacceptable levels of blood cell trauma. We have adapted technology from our intravenous respiratory assist catheter to accomplish these objectives [24, 25]. Using an array of rotating impellers within an annular hollow fiber membrane bundle, high fluid velocities, and improved blood flow distribution enhances gas exchange. This paper reports on the design and bench testing of an ultra-low-flow ECCO_2_R device (ULFED) utilizing the rotating impeller concept. In vitro gas exchange and hemolysis were evaluated.

## Methods

### Device description

**Fig. 1:**
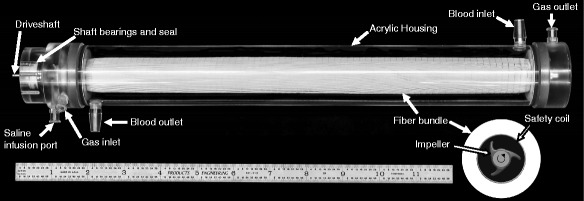
ULFED prototype and cross-sectional schematic showing arrangement of impellers surrounded by safety coil and annular fiber bundle

**Fig. 2:**
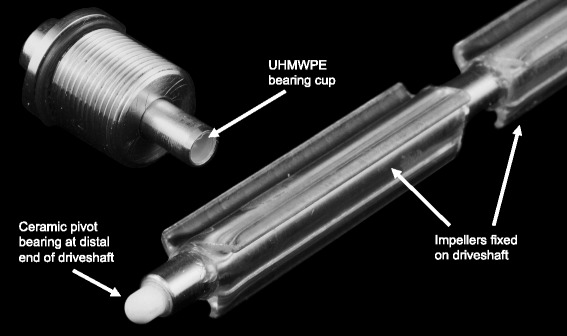
Distal end of ULFED driveshaft and pivot bearing

The ultra-low-flow ECCO_2_R device (ULFED) (Fig. 1) contains six rotating impellers fixed on a rigid stainless steel driveshaft (3/16 in. diameter [4.76 mm]). A stainless steel safety coil surrounding impellers (diameter 14.4 mm) protects a 30-cm-long polypropylene (PP) fiber bundle (300 μm diameter, x30-240; Membrana Celgard, Wuppertal, Germany) with total surface area 0.42 m^2^. The bundle/impeller assembly is housed in cylindrical acrylic tubing (inner diameter 1.375 in. [34.9 mm]) with 1/4 in [6.35 mm] inflow/outflow ports for a total priming volume of 240 mL. Impellers (Fig. 2) were fabricated from a hydrophobic epoxy resin (Watershed XC11122; DSM Somos, Sittard) using stereolithography (SLA). Impellers measured 4 mm in length with a maximum outer diameter of 11.7 mm. Impellers are designed to only generate flow radially in/out of the surrounding fiber bundle to avoid perturbing circuit blood flow rates.

The impeller drive shaft extends out of the blood pathway and is sealed (400054; SKF, Gothenburg, Sweden) and supported by bearings (Ceramic R3; Ortech, Inc., Sacramento, CA). An external DC brushless servomotor (4490 H 048B; MicroMo Electronics, Inc., Clearwater, FL) drives shaft rotation. Saline is continuously infused along the shaft at 30 mL/h to lubricate and protect the seal and bearing from blood backflow up the driveshaft. Heparin was added to the saline infusion (20 U/mL) to maintain anticoagulation levels in the blood for consistency across test circuits. The shaft is supported distally using a custom pivot bearing (ceramic pin (MSC Industrial Supply, Melville, NY) nested in an ultra-high molecular weight polyethylene cup (UHMWPE; Orthoplastics, Lancashire, UK)) shown in Fig. 2.

### Gas exchange

**Fig. 3:**
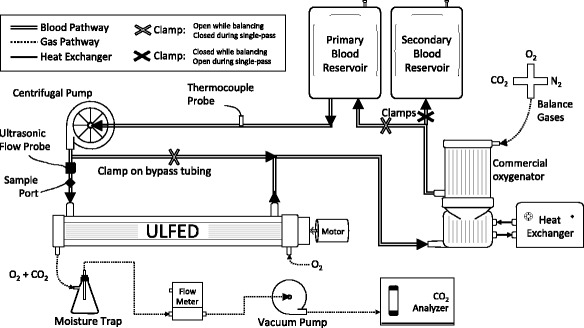
Recirculating blood loop used to evaluate in vitro gas exchange performance

CO_2_ removal performance of the ULFED prototype was evaluated in a single-pass flow loop (Fig. 3) at a hemodialysis blood flow rate of 250 mL/min. The evaluations followed ISO 7199:2009 standards for gas exchange testing in blood oxygenators [26]. Filtered and heparinized bovine blood (20 U/mL) was collected fresh from the slaughterhouse the day of testing. The fluid circuit consisted of a centrifugal blood pump (BPX-80; Medtronic, Minneapolis, MN), a commercial oxygenator (Affinity NT; Medtronic, Minneapolis, MN), two blood reservoirs connected in parallel, and the ULFED. Blood continuously recirculated at 4500–5500 mL/min while gas tensions were balanced by the commercial oxygenator to normocapnic venous conditions (pCO_2_ = 45 ± 5 mmHg) using a N_2_/CO_2_/O_2_ gas mixture. Blood temperature was maintained at 37 ± 1 °C with a heat exchanger integrated into the commercial oxygenator. Flow recirculated only to and from the primary reservoir during balancing, while secondary reservoir tubing remained clamped. Gas levels in the recirculating loop were monitored with a blood gas analyzer (RapidPoint 405; Siemens, Erlangen, Germany) until venous conditions were reached. The loop was then converted to single-pass mode for data collection by diverting flow to the secondary reservoir and clamping the bypass tubing in parallel to the ULFED. Measurements were collected at rotation speeds from 0 to 5000 RPM once all measured parameters remained stable for ≥ 2 min. The order of tested rotation speeds was randomized, and a minimum of two measurements were collected at each rotation speed. Blood flow rate was continuously monitored with an ultrasonic flow probe (Transonic Systems, Ithaca, NY).

Pure O_2_ sweep gas was pulled through fibers counter-current to blood flow at 8.0 L/min by a sealed vacuum pump (N811 KV.45P; KNF Neuberger, Trenton, NJ) and was regulated with a thermal mass flow controller (GR-116-1-A-PV-O_2_; Fathom Technologies, Georgetown, TX). The fraction of CO_2_ in outlet sweep gas $\left({F}_{{\mathrm{CO}}_2}\right)$ was measured by a gaseous CO_2_ analyzer (WMA-4; PP Systems, Amesbury, MA) and was used to calculate total CO_2_ removal (${V}_{{\mathrm{CO}}_2}$) together with the STP-corrected sweep gas flow rate $\left({Q}_{\mathrm{OUT}}^{\mathrm{STP}}\right)$ according to Eq. 1.

1 $$ {V}_{\mathrm{C}{\mathrm{O}}_2}={Q}_{\mathrm{O}\mathrm{UT}}^{\mathrm{STP}}{F}_{\mathrm{C}{\mathrm{O}}_2} $$

${V}_{\mathrm{C}{\mathrm{O}}_2}$ was normalized to our target inlet pCO_2_ of 45 mmHg to reduce variability in measurements associated with small fluctuations in gas inlet conditions (± 5 mmHg) according to Eq. 2.

2 $$ {V}_{\mathrm{C}{\mathrm{O}}_2}^{\ast }={V}_{\mathrm{C}{\mathrm{O}}_2}\times \frac{45\mathrm{mmHg}}{pC{O}_2^{\mathrm{INLET}}} $$

$pC{O}_2^{\mathrm{INLET}}$was measured from a fresh blood sample immediately prior to each data point. Three identical ULFED prototypes were fabricated for repeatability testing, but gas pathway failure in one device limited gas exchange testing to two devices. Total ULFED gas exchange is reported as average and standard deviation of the CO_2_ removal rates of both prototypes at each rotation speed.

### In vitro hemolysis testing

Filtered and heparinized bovine blood (20 U/mL) was collected fresh from the slaughterhouse the day of testing per ASTM standards (F1841–97) [27]. The gas exchange loop was modified for hemolysis testing by removing the bypass tubing parallel to the ULFED, the commercial oxygenator, the secondary reservoir, and the ULFED gas pathway components. The ULFED was evaluated in two circuits so that overall hemolysis reflected that of clinical setups. A reasonable cannula for ECCO_2_R at the target 250 mL/min of blood flow (13 Fr Avalon Elite DLC 10013; Maquet, Rastatt, Germany) and a pediatric centrifugal pump (PediMag; Thoratec, Pleasanton, CA) were selected for the ULFED “standard circuit”. Blood (1000 mL) was continuously recirculated for 3 h. The reservoir was submerged in a heated water bath to maintain a circuit temperature of 37° ± 1 °C. ULFED rotation was set to the minimum speed necessary where CO_2_ removal did not differ significantly from the maximum rate achieved. The second ULFED circuit (“dialysis configuration”) evaluated performance using a hemodialysis controller roller pump (Prisma; Baxter, Deerfield, IL) and cannula. A larger bore 14-Fr, 15-cm dialysis cannula (AK-22142-F; Teleflex, Morrisville, NC) was used in the second circuit due to availability of parts recommended for the target blood flows.

A control circuit (“Minimax”) was tested to evaluate ULFED hemolysis against an approved low-flow blood oxygenator (Minimax Plus; Medtronic, Minneapolis, MN). Blood flow in the control loop was maintained at the minimum rate necessary (1250 mL/min) to match ULFED CO_2_ removal performance according to the manufacturer [28]. Pump (BP-50; Medtronic, Minneapolis, MN) rotation speed in the loop was maintained at 2100–2200 RPM against 180 mmHg to simulate inclusion of cannula recommended for use at the target blood flows (14 Fr Biomedicus 96820-014 venous, 12 Fr Biomedicus 96820-012 arterial) [29, 30]. Pressure against the pump was adjusted using a Hoffman clamp on ULFED outlet tubing and was continuously monitored with a differential fluid pressure transducer (PX771-025DI; Omega Engineering, Inc., Stamford, CT) across the pump. All other components and conditions were consistent between circuits. All three ULFED prototypes fabricated for gas exchange testing were evaluated for hemolysis in both circuit configurations, as the gas pathway failure observed in one prototype did not interfere with hemolysis testing.

Samples were drawn every 30 min to measure hematocrit (HCT) and plasma-free hemoglobin (pfHb). Plasma was isolated from whole blood in two centrifuge spins (15 min at 0.8*g*, 10 min at 7.2*g*), and absorbance at 540 nm was measured spectrophotometrically (Genesys 10S UV-Vis; Thermo Scientific, Waltham, MA). PfHb concentration was calculated from absorbance using a standard curve developed from a linear-fit of serially diluted whole blood with 100% hemolysis versus absorbance [31].

The normalized index of hemolysis (NIH) was calculated for circuit comparisons:

3 $$ \mathrm{NIH}\ \left(g/100L\right)=\Delta pfHb\times V\times \frac{100-\mathrm{HCT}}{100}\times \frac{100}{\varDelta t\times Q} $$

Where NIH = normalized index of hemolysis in grams of hemoglobin released into the blood per 100 L of flow through the circuit (g/100 L); Δ*pfHb* = increase in pfHb over the sampling time interval (g/L); *V* = circuit volume (L); HCT = hematocrit (%); Δ*t* = sampling time interval (min); *Q* = average blood flow rate (L/min). A time-of-therapy normalized index was also calculated, since the NIH equation does not reflect total hemolysis returned to a patient in the context of treatment duration. Flow rate normalization in the NIH equation is eliminated in the new therapeutic index of hemolysis (TIH) calculation to indicate the total grams of hemoglobin released to the blood per 100 min of therapy (g/100 min):

4 $$ \mathrm{TIH}\ \left(g/100\min \right)=\Delta pfHb\times V\times \frac{100-\mathrm{HCT}}{100}\times \frac{100}{\varDelta t} $$

### Statistics

All statistical comparisons were conducted in SPSS (IBM, Armonk, NY). A one-way ANOVA with Tukey HSD post hoc testing was used to compare removal rates at each RPM after data satisfied assumptions of homogeneity of variance, normality, and independence. Comparisons were used to identify the minimum speed necessary to achieve statistically equivalent performance to the maximum CO_2_ removal rate. The determined rotation speed was used for subsequent hemolysis testing. Mean NIH values were compared using a one-way ANOVA with Tukey HSD post hoc test after satisfying relevant assumptions. TIH data violated the assumption of homogeneity of variance via Levene’s test, and means were compared with Welch’s *F* test. Subsequent Games-Howell post hoc tests were used for between-group comparisons of means. All comparisons of means were considered significant at the level *p* < 0.05.

## Results

### In vitro gas exchange

**Fig. 4:**
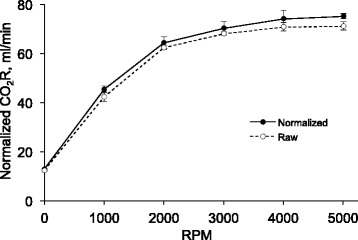
Raw (${V}_{\mathrm{C}{\mathrm{O}}_2}$) and normalized (${V}_{\mathrm{C}{\mathrm{O}}_2}^{\ast }$) CO_2_ removal rates versus rotation speed. Error bars represent one standard deviation of measured removal rates

Figure 4 shows the raw and normalized CO_2_ removal rates of the ultra-low-flow CO_2_ removal device (ULFED) as a function of impeller rotational speed. A sharp increase in CO_2_ removal occurred between 0 and 2000 RPM before subsequently leveling off at higher rotation speeds. A maximum normalized CO_2_ removal rate of 75.1 ± 1.1 mL/min was achieved at 5000 RPM. Normalized performance at 4000 RPM did not differ significantly from the maximum rate however (74.1 ± 3.3 mL/min, *p* = 0.99).

### In vitro hemolysis

**Fig. 5:**
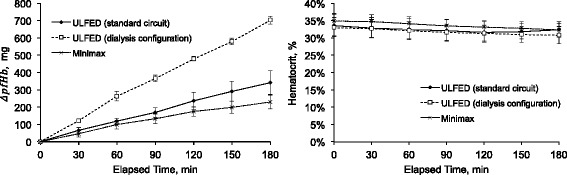
Average change in total plasma free hemoglobin (pfHb) versus baseline (left) and average hematocrit (right) at 30 min sampling intervals during in vitro hemolysis tests. Error bars represent one standard deviation at each time-point between tests for each circuit

Measured rates of pfHb accumulation were highly linear over testing periods as shown in Fig. 5 (Δ*pfHb* versus elapsed time *R* ^2^ > 0.95 in all tests). Table 1 shows the calculated hemolysis indices for the ULFED (at 4000 PRM) and the control device. NIH values for the standard-ULFED circuit (0.78 ± 0.19 g/100 L), dialysis-ULFED circuit (1.55 ± 0.03 g/100 L), and the control circuit (0.11 ± 0.01 g/100 L) each differed significantly from one another (ANOVA *p* < 0.001, all group-wise comparisons *p* < 0.001). The TIH value of the standard-ULFED (0.190 ± 0.041 g/100 min) did not differ significantly from the control circuit (0.123 ± 0.013 g/100 min; Welch’s test *p* < 0.001, group-wise *p* = 0.169). The hemolysis using dialysis circuit components (0.386 ± 0.010 g/100 min) was significantly greater than both other test groups (each *p* < 0.05). Average hematocrit at each sampling interval is shown in Fig. 5 (right). Hematocrit decreased by ~ 1.5–2.5% from baseline over test periods, which is consistent with dilution due to saline infusion.

**Table 1:** Summary of in vitro hemolysis testing

Test device	Blood flow	TIH		NIH	
	(mL/min)	(g/100 min)		(g/100 L)	
ULFED (standard circuit)	250	0.190	±0.041	0.775	±0.186*
ULFED (dialysis configuration)	250	0.386	±0.010*	1.551	±0.025*
Minimax	1250	0.123	±0.013	0.105	±0.012*

*Significant at *p* < 0.05 versus all other devices. *TIH* therapeutic index of hemolysis. *NIH* normalized index of hemolysis

## Discussion

Supplementing respiration by removing CO_2_ independent of the lungs can improve outcomes for patients at risk of requiring or already receiving invasive mechanical ventilation. We developed the ultra-low-flow ECCO_2_R device (ULFED) to operate at blood flow rates consistent with renal hemodialysis to simplify circuit management and minimize invasiveness of CO_2_ removal. In vitro CO_2_ removal rates up to 74 mL/min at 4000 RPM were achieved by the ULFED with minimal cell trauma (therapeutic index of hemolysis, TIH = 0.19 g/100 min) at blood flows consistent with dialysis (250 mL/min).

CO_2_ removal systems used in conjunction with non-invasive or protective ventilation strategies have been shown to correct pCO_2_ and pH in hypercapnic patients with removal rates equivalent to ~ 25–35% of the metabolic CO_2_ production (~ 200–250 mL/min) [22, 23]. CO_2_ removal at these levels prevented intubation in patients with ae-COPD failing or unresponsive to non-invasive ventilation [14, 16, 17, 32]. Partial respiratory assistance has also aided weaning from ventilation [14, 33, 34] and allows reduction of ventilator tidal volumes to ultra-protective levels (3–4 mL/kg) [21, 35, 36]. The ULFED exceeded these CO_2_ removal rates by eliminating ~ 30–37% of the metabolic CO_2_ production at normocapnic test conditions (inlet pCO_2_ = 45 mmHg). Gas exchange will also increase proportionally with pCO_2_ in hypercapnic patients, where CO_2_ removal up to 50% or more of metabolic production can be required.

Efficient gas exchange in the ULFED minimizes necessary fiber surface area and enables clinically significant CO_2_ removal rates at hemodialysis blood flows. Pump-less arteriovenous CO_2_ removal (AVCO_2_R) requires dual cannulation (13–19 Fr) for circuit flows of 600–2000 mL/min that is shunted between the femoral artery and vein through a 1.3-m^2^ oxygenator [36–38]. A newer integrated pump-oxygenator system uses a rotating core to generate active mixing to improve gas exchange up to 60% with a 0.59-m^2^ bundle [39]. Comparatively lower flows (350–500 mL/min) are possible in the simplified veno-venous circuit, but CO_2_ removal decreases with blood flow (~ 50 mL/min at 300 mL/min blood flow) and connection requires 15.5-Fr cannulation [4, 39]. Developing systems combine existing oxygenators with dialysis controllers targeting even lower flows (200–300 mL/min) to minimize cannulation invasiveness (≤ 14 Fr). These systems utilize larger surface area gas exchangers (≥ 1 m^2^) to improve performance [32, 40] or target lower CO_2_ removal using smaller pediatric oxygenators (40–55 mL/min with a 0.3-m^2^ bundle in pigs with PaCO_2_ > 80 mmHg) [41]. Approaches to enhance CO_2_ removal such as bicarbonate dialysis [42], blood acidification [43], electrodialysis [44], plasma recirculation [45], and fiber enzyme coatings [46] are also being explored to reduce necessary blood flows for treatment.

The rotating impellers in the ULFED enhance gas transfer by generating an “active mixing” effect in the fiber bundle that improves convective mixing at gas exchange surfaces [24, 25, 47, 48]. Computational simulations have indicated development of continuously recirculating flow pathways in/out of the fiber bundle with impeller mixing [25]. Blood is pumped radially outward through the bundle by impellers, then pulled back into the bundle toward low-pressure regions in the gaps between impellers before converging onto the impeller blade and cycling through the bundle again. Increasing flow velocity past gas exchange surfaces is a well-established mechanism for improving transfer efficiency by diminishing the thickness of the surface diffusive boundary layer [49]. This facilitates replenishment of gases to the membrane surface to maximize the concentration gradients spanning fiber walls. Recirculating flow also maintains a high level of washing in the bundle that eliminates regions of stagnation where thrombus formation may otherwise occur at low blood flows.

The measured rate of hemolysis in the standard-ULFED circuit was comparable to a clinically approved oxygenator circuit. Two indices of red cell trauma are reported here that indicate the rate of pfHb accumulation over time, the key difference being how time is reported. A major limitation of the NIH calculation is that hemolysis is normalized for blood flow rate, but operating flow rate is ultimately irrelevant. Two systems intended for use at 5000 mL/min versus 250 mL/min that cause equivalent rates of total cell damage would differ in NIH by a factor of 20, despite returning an equal number of pfHb species to a patient. As a result the NIH calculation is bias against low-flow devices. The TIH calculation removes the flow rate normalization and provides a clinically relevant time-of-therapy rate of hemolysis. The limitation of both indices however is that no reliable benchmark threshold values have been validated for low-flow devices against in vivo performance to our knowledge. More information or in vivo testing is therefore necessary to make conclusions regarding acceptability of the dialysis-ULFED performance. No difference in hemolysis was observed between the control and standard-ULFED circuits, so we expect in vivo hemolysis to be acceptable in this configuration.

Anticoagulation of saline infused to the ULFED may have clinical implications with extended use. Heparin levels in the saline infusion line were chosen primarily to avoid dilution of circuit anticoagulation for consistency between tests. Approved blood pumping devices utilizing saline-lubricated seals anticoagulate infusion lines at higher rates [50], while others do not require anticoagulation [51]. Elimination or minimization of local anticoagulation from the ULFED will be investigated in future prototypes utilizing a saline infusion line.

## Conclusions

Evidence continues to grow that ECCO_2_R can effectively prevent intubation, facilitate earlier extubation, or allow reduction of ventilator settings in hypercapnic respiratory failure. The ULFED eliminates clinically significant levels of CO_2_ from blood with acceptable hemolysis at hemodialysis blood flows, making minimally invasive dialysis connection strategies and simplified management possible for ECCO_2_R. Future work may focus on in vivo validation of benchtop performance or improvements to the ULFED aimed at simplifying the design, such as sealing the blood compartment with a magnetically coupled driveshaft that would obviate the driveshaft seal and saline infusion.

## Abbreviations

ECCO_2_R: Extracorporeal carbon dioxide removal
ECMO: Extracorporeal membrane oxygenation
NIH: Normalized index of hemolysis
pfHb: Plasma-free hemoglobin
TIH: Therapeutic index of hemolysis
ULFED: Ultra-low-flow ECCO_2_R device
